# Trimester-Specific Associations Between Maternal Fatty Acid Ratios and Cognitive and Executive Functioning in 4-Year-Old Children

**DOI:** 10.3390/nu18213514

**Published:** 2026-10-26

**Authors:** Xiruo Kou, Josefa Canals, Victoria Arija

**Affiliations:** 1 Nutrition and Mental Health (NUTRISAM) Research Group, Nutrition and Public Health Unit, Universitat Rovira i Virgili, C/Sant Llorenç 21, 43204 Reus, Spainjosefa.canals@urv.cat (J.C.); 2 Institut d’Investigació Sanitaria Pere Virgili (IISPV), 43204 Reus, Spain

**Keywords:** early-life nutrition, fatty acid balance, gestational window, neurodevelopment, children

## Abstract

**Purpose**: Early prenatal fatty acid balance is vital for brain development, yet trimester-specific effects on long-term neurodevelopment remain unclear. This study examined associations between maternal omega-6/omega-3 (*n*-6/*n*-3) ratios in the first and third trimesters and child neurodevelopment at age four. **Methods**: In the ECLIPSES cohort (*n* = 223), maternal serum *n*-3 and *n*-6 fatty acids were measured at 12 and 36 gestational weeks. At age four, children’s cognitive and executive function were assessed using the Wechsler Preschool and Primary Scale of Intelligence (WPPSI), and the Behavior Rating Inventory of Executive Function (BRIEF)—Preschool Version, respectively. Multivariable regression models were adjusted for sociodemographic and lifestyle factors. **Results**: A higher *n*-6/*n*-3 ratio in the first trimester was significantly associated with poorer executive functioning, as reflected by higher BRIEF scores indicating greater difficulties in inhibition (b = 2.45, *p* = 0.002), working memory (b = 1.87, *p* = 0.030), planning/organization (b = 2.20, *p* = 0.004), and the Global Executive Composite (b = 1.96, *p* = 0.006). Higher first-trimester ratios were also associated with lower WPPSI working memory performance (b = −2.14, *p* = 0.013), while general cognitive abilities were largely unaffected. **Conclusions**: An elevated maternal *n*-6/*n*-3 ratio in early pregnancy is associated with poorer executive function in preschool children, whereas evidence linking these ratios to general cognition remains limited. These findings indicate that associations between fatty acid balance and executive function were mainly observed during the first trimester.

## 1. Introduction

Early brain development is highly sensitive to the intrauterine nutritional environment [1]. Long-chain polyunsaturated fatty acids (LCPUFAs), particularly docosahexaenoic acid (DHA), an omega-3 fatty acid (*n*-3) and arachidonic acid (ARA), an omega-6 fatty acid (*n*-6), are essential for neurogenesis and synaptogenesis [1,2]. As the fetus depends on maternal supply via placental transfer, maternal fatty acid status has lasting implications for offspring neurodevelopment [3]. Given that fetal brain development occurs in distinct stages, the timing of maternal exposure may differentially influence later neurodevelopmental outcomes [3,4].

The prior literature has primarily focused on *n*-3 fatty acids in isolation [5,6]. While some evidence suggests that increasing maternal *n*-3 levels can benefit neurodevelopment [7], findings remain inconsistent. High-dose DHA supplementation may not improve long-term behavioral functioning and can even lead to heterogeneous outcomes depending on timing and dosage [5,8,9,10]. This underscores that focusing solely on *n*-3 levels provides an incomplete picture. A key factor contributing to these inconclusive results is the oversight of *n*-6 fatty acids and their interaction with *n*-3. Because *n*-3 and *n*-6 LCPUFAs compete for the same enzymatic pathways, their relative balance is biologically more meaningful than absolute levels [11,12]. This physiological imbalance has clinical implications, research has shown that a lower prenatal *n*-3/*n*-6 ratio, reflecting a higher *n*-6 contribution, is associated with higher scores in internalizing and externalizing problems in children at age six [13,14]. Similarly, an elevated *n*-6/*n*-3 ratio has been consistently linked to specific neurodevelopmental challenges, such as a higher risk of ADHD-related symptoms, including attentional and hyperactivity issues across different pediatric populations [15,16]. Furthermore, this imbalance was also evidenced by poorer infant motor development in our cohort’s previous analysis [3], suggesting that the *n*-6/*n*-3 ratio may be a more sensitive predictor of multi-domain neurodevelopmental outcomes than the levels of *n*-3 fatty acids alone. It is important to note that while dietary *n*-6/*n*-3 ratios reflect external nutritional intake, circulating serum fatty-acid ratios serve as an integrated biomarker of the maternal metabolic and lipid milieu, shaped by both diet and endogenous metabolism.

Several critical research gaps remain. First, a limitation lies in the incomplete understanding of maternal fatty acid (FA) status across gestation. Most studies rely on single-time-point measurements and fail to capture the dynamic physiological shifts in maternal FA concentrations, which are driven by adipose tissue mobilization and placental transfer [16,17]. Second, while cognitive and behavioral domains are equally vital for child neurodevelopment [18], most existing research focusing on multiple trimesters tends to evaluate only one of these domains, leading to a fragmented understanding of overall development. Third, current longitudinal evidence is predominantly limited to outcomes within the first two years of life. While our previous cohort analysis provided preliminary evidence linking prenatal fatty acid ratios to motor development in infants at approximately 54 days postpartum [3], these early assessments may not capture the later emergence and consolidation of higher-order cognitive processes. Core executive functions, including inhibitory control and working memory develop progressively and become more robust as the prefrontal cortex matures during the preschool years [19,20].

Thus, this study aimed to examine the associations between maternal *n*-6/*n*-3 ratio in the first and third trimesters and child neurodevelopment at age four. Cognitive abilities were assessed using the Wechsler Preschool and Primary Scale of Intelligence (WPPSI), and executive functions were evaluated using the Behavior Rating Inventory of Executive Function–Preschool Version (BRIEF-P). By incorporating trimester-specific data, this research clarifies whether fatty acid balance in early versus late pregnancy is differentially associated with later cognitive and parent-reported executive outcomes.

## 2. Methods

### 2.1. Study Design and Participants

This prospective follow-up study involved pregnant women from their first trimester through early postpartum as part of the ECLIPSES cohort, a community-based randomized controlled trial in Spain. A total of 791 women were enrolled before 12 weeks of gestation and assessed at 12, 24, and 36 weeks of pregnancy. Children were assessed at approximately 54 days after birth (*n* = 336) and at 4 years of age (*n* = 223). Eligibility required participants to be over 18, have a singleton pregnancy under 12 weeks, and understand Spanish or Catalan. Women with anemia (Hb < 110 g/L), multiple pregnancies, egg protein allergy, or severe pre-existing conditions (e.g., immunosuppression, malabsorption, diabetes, cancer, liver disease) were excluded. More detailed information can be found elsewhere [21].

A total of 791 pregnant women were initially enrolled in the ECLIPSES cohort. During the follow-up, 257 participants were excluded or lost due to various reasons, such as voluntary withdrawal, miscarriage, or not meeting the inclusion criteria, leaving 534 women who completed the study. Among them, 450 participants had available maternal LCPUFA measurements during pregnancy and were included in the intermediate analytical subsample. Finally, after excluding those without available neurodevelopmental assessments at the 4-year postpartum visit, a total of 223 mother–infant pairs comprised the final analytic sample. The study flow chart was presented in Figure S1.

### 2.2. Exposure Measurements

Maternal fasting blood samples were collected, centrifuged, and serum was stored at −80 °C until analysis. Proteins were precipitated from serum using methanol containing an internal standard. Long-chain polyunsaturated fatty acids (PUFAs) including eicosapentaenoic acid (EPA), DHA, and ARA were analyzed as fatty acid methyl esters (FAMEs) via gas chromatography–mass spectrometry (GC-MS) and followed by purification via liquid–liquid extraction. Separation was performed on a high-polarity HP-88 column using gradient elution or a temperature program. Detection was carried out on a GC-MS system (7890A GC coupled to a QqQ 7000 Series; Agilent Technologies, Santa Clara, CA, USA) in Selected Ion Monitoring (SIM) mode. Total *n*-3 PUFAs were determined as the sum of eicosapentaenoic acid (EPA, 20:5*n*-3) and docosahexaenoic acid (DHA, 22:6*n*-3). Total *n*-6 PUFAs were determined as the sum of linoleic acid (LA, 18:2*n*-6), dihomo-γ-linolenic acid (DGLA, 20:3*n*-6), and arachidonic acid (ARA, 20:4*n*-6). The total *n*-6/*n*-3 ratio was calculated as total *n*-6 PUFAs divided by total *n*-3 PUFAs. Detailed analytical procedures, instrument conditions, and the complete list of identified fatty acids are provided in the Supplementary Information (see Supplementary Methods).

### 2.3. Outcome Measurements

Executive functioning was evaluated via the Spanish parent-completed BRIEF-P [22], which has adequate psychometric properties [23]. This 36-item tool assesses behaviors over the previous six months, yielding five clinical subscales: inhibition (INH), shifting (SHI), emotional control (EC), working memory (WM), and planning/organization (PO). These form three composite indices: Inhibitory Self-Control Index (ISCI), Flexibility Index (FI), and Emergent Metacognition Index (EMI), plus a Global Executive Composite (GEC). T-scores (mean = 50, SD = 10) were used, with higher scores indicating greater executive difficulties.

Simultaneously, trained psychologists assessed cognitive abilities using the Spanish WPPSI-IV. Primary composite scores included the Verbal Comprehension Index (VCI), Fluid Reasoning Index (FRI), Working Memory Index (WMI), Processing Speed Index (PSI), and Full-Scale IQ (FSIQ). Secondary indexes, such as the Vocabulary Acquisition Index (VAI), Nonverbal Index (NVI), and General Ability Index (GAI), were also calculated. Standardized scores (mean = 100, SD = 15) were used, with higher scores indicating better cognitive performance [24].

### 2.4. Covariates Measurements

At the time of enrollment, mothers participated in in-person interviews using structured questionnaires to collect baseline information. Data gathered covered demographic characteristics, Body Mass Index (BMI, kg/m^2^) computed at the first and third trimesters, smoking habits during the pregnancy (smokers and non-smokers), and adherence to the Mediterranean diet (MedDiet) estimated in the first and third trimesters using Food Intake Frequency Questionnaire [25]. Information on the education level and occupational status of the participants and their partners was collected using the Catalan Classification of Occupations, and the family socioeconomic status was calculated as low, medium or high, according to the Hollingshead Index [26]. An approximation to parental intellectual ability was determined using the Matrix Reasoning component of the Wechsler Adult Intelligence Scale–III (WAIS-III) [27]. Child-related variables comprised gestational age at birth recorded in health cards, along with sex and feeding method reported by mothers. Furthermore, children’s dietary quality at 4 years of age was measured using the Spanish Diet Quality Index (SDQI), which follows the nutritional guidelines issued by the Spanish Society of Community Nutrition [28].

### 2.5. Statistical Analysis

Missing values were addressed through multiple imputation using chained equations (MICE) under the missing-at-random (MAR) assumption. The imputation procedure incorporated exposures, covariates, and outcomes as predictors to increase the validity of the imputations and maintain consistency with the analytic framework. Outcome data were not imputed due to the risk of introducing bias in longitudinal neurodevelopmental outcomes; therefore, analyses were limited to participants with observed outcome measurements. Given the minimal proportion of missing data across all covariates (all ≤4.0%), five imputed datasets were generated. Regression models were estimated within each dataset separately, and the estimates and standard errors were then combined according to Rubin’s rules to produce pooled effect estimates with 95% confidence intervals [29]. Exposure variables were natural log-transformed and subsequently standardized (z-scored) prior to analysis to reduce skewness and improve model fit, meaning that regression coefficients reflect changes in outcomes per 1-SD increase in the log-transformed fatty acid ratios. Multivariable regression models were adjusted for mother or parents’ covariates: age, time-specific maternal BMI (specifically, early/pre-pregnancy baseline BMI for first-trimester exposure models, and concurrent third-trimester maternal BMI for third-trimester exposure models to account for normal gestational weight progression), family social class, smoking status, MedDiet score, parental IQ (included in the WPPSI models to account for the heritability of direct cognitive test performance, but omitted from the BRIEF-P models to avoid potential reporter bias and over-adjustment in parent-reported behavioral assessments); and infant covariates: gestational age, child sex, type of feeding, and SDQI score. To avoid structural bias such as collider bias and over-adjustment, post-exposure variables that occur after the prenatal exposure period (such as infant feeding practices and subsequent child diet) were excluded from the primary models, as true confounders must precede the exposure. Instead, models additionally incorporating these post-exposure factors were evaluated as sensitivity analyses to examine the robustness of the primary findings. To distinguish primary hypothesis testing from exploratory analyses, the GEC from the BRIEF-P and FSIQ from the WPPSI-IV were pre-specified as primary outcomes, while individual subscales and clinical indices were treated as secondary endpoints. To control for potential false discoveries arising from multiple comparisons, the Benjamini–Hochberg False Discovery Rate (FDR) procedure was applied within outcome families for each trimester. All statistical analyses were performed using R (version 4.4.1). The statistical significance level was set at α = 0.05 and all statistical tests were two-sided.

## 3. Results

### 3.1. Study Population Characteristics

No differences were observed between included and excluded participants (Supplementary Table S1). Table 1 presented the characteristics of the study population (n = 223). Maternal BMI increased from the first to the third trimester (25.13 ± 4.64 vs. 29.20 ± 4.38, *p* < 0.001). ARA/DHA decreased, whereas ARA/EPA and *n*-6/*n*-3 ratio increased during pregnancy (all *p* < 0.001). Most mothers were non-smokers (86.1%), and 79.4% of infants were breastfed. The mean gestational age was 39.94 weeks (SD 1.14), and the mean SDQI score of infants at 4 years was 61.78 (SD 10.17), indicating an average diet quality. Table 2 illustrated the neurodevelopment scores of the children. In the BRIEF-P scales, mean scores ranged from 49.63 (SD 10.34) to 50.84 (SD 10.08), with a mean GEC score of 50.40 (SD 9.89). In the WPPSI scales, the mean FSIQ was 102.25 (SD 11.68), and index scores ranged from 95.71 (SD 12.63) for PSI to 104.93 (SD 13.35) for VCI. Descriptively, sex differences were significant across most domains, with males scoring higher on BRIEF-P scales and females scoring higher on WPPSI indices, including FSIQ.

### 3.2. Associations Between Prenatal Fatty Acid Ratios and Executive Functioning Outcomes

Potential interactions between child sex and fatty acid ratios were assessed for all outcomes. Since no significant interactions were observed (Supplementary Table S2), all subsequent analyses were performed in the total population without stratification by sex. A higher prenatal *n*-6/*n*-3 ratio in the first trimester was associated with higher BRIEF scales scores for INH (b = 2.45, 95% CI: 0.93–3.96, *p* = 0.002), WM (b = 1.87, 95% CI: 0.19–3.55, *p* = 0.030), and PO (b = 2.20, 95% CI: 0.72–3.69, *p* = 0.004). Positive associations were also observed for the ISCI (b = 1.89, 95% CI: 0.44–3.34, *p* = 0.011), EMI (b = 1.99, 95% CI: 0.43–3.55, *p* = 0.014), and GEC (b = 1.96, 95% CI: 0.58–3.34, *p* = 0.006). Furthermore, the associations persisted after FDR correction. No significant associations were found for the third trimester (Figure 1).

For the ARA/DHA ratio, a higher ratio in the first trimester was associated with higher EC scores (b = 2.09, 95% CI: 0.47–3.72, *p* = 0.012) and ISCI scores (b = 2.08, 95% CI: 0.39–3.77, *p* = 0.017). In the third trimester, a higher ARA/DHA ratio was positively associated with EC scores (b = 2.17, 95% CI: 0.27–4.07, *p* = 0.027) and FI scores (b = 2.22, 95% CI: 0.57–3.87, *p* = 0.009). In addition, the ARA/EPA ratio in the first trimester was positively associated with INH scores (b = 1.58, 95% CI: 0.00–3.17, *p* = 0.050) and ISCI scores (b = 1.78, 95% CI: 0.09–3.47, *p* = 0.039), whereas no significant associations were observed for the third trimester (Figure 2). However, these associations were attenuated and no longer reached statistical significance after FDR adjustment. Detailed results were presented in Supplementary Tables S3–S5.

### 3.3. The Association Between Prenatal Fatty Acid Ratios and Cognitive Outcomes

A higher prenatal *n*-6/*n*-3 ratio in the first trimester was associated with lower WMI (b = −2.14, 95% CI: −3.82 to −0.46, *p* = 0.013). In the third trimester, *n*-6/*n*-3 ratio was positively associated with FRI (b = 2.66, 95% CI: 0.08–5.23, *p* = 0.044). However, these associations did not withstand FDR correction for multiple testing (Figure 3). Furthermore, no associations were found between prenatal ARA/DHA or ARA/EPA ratios and WPPSI-IV scores (Figure 4). Complete results were shown in Supplementary Tables S6–S8. Furthermore, sensitivity analyses adjusting for post-exposure factors (such as infant feeding practices and subsequent child diet) showed similar results (Figures S2–S5), confirming the robustness of our primary findings.

## 4. Discussion

In this study, higher prenatal *n*-6/*n*-3 ratio during the first trimester was associated with poorer executive functioning among children via BRIEF-P, particularly in inhibition, WM, and planning/organization. These associations were weaker or absent during the third trimester. Notably, in the first trimester, WMI as measured by the WPPSI, a direct, performance-based measure, was the only cognitive domain associated with higher prenatal *n*-6/*n*-3 ratio, while no associations were observed for the other cognitive domains. Overall, these findings highlight the potential importance of early gestational fatty acid balance for the development of executive functions.

Our results align with prior studies linking elevated prenatal *n*-6/*n*-3 ratios to adverse neurobehavioral outcomes, such as increased ADHD risk at age 7 [14]. Given that children with ADHD typically exhibit executive difficulties, including challenges in working memory, inhibitory control, and emotional regulation, their findings align with the poorer functioning observed in our cohort. In contrast, while it has also been found that greater concentrations of DHA decreased the risk for emotional symptoms, previous reported associations between a higher *n*-6/*n*-3 ratio and hyperactivity/inattention symptoms did not reach statistical significance [15]. Notably, while the WPPSI showed significant results only for the WMI, the BRIEF-P revealed broader difficulties across multiple executive domains. This discrepancy likely reflects the nature of the assessments. The WPPSI WMI is a performance-based measure of maximal cognitive capacity in a structured setting [24], whereas the BRIEF-P, based on parent reports, captures behavioral manifestations of executive functioning in daily life [22]. The consistency of the WM findings across both direct and observer-report measures suggests that working memory may be particularly sensitive to early nutritional programming, while the broader BRIEF-P results capture more pervasive behavioral regulation challenges in real-world environments. When interpreting the stronger associations observed for the BRIEF-P compared to the WPPSI-IV, several factors should be considered. First, the two instruments capture different contexts: everyday behavioral regulation versus standardized cognitive performance. Second, potential reporter-related variance, residual confounding, and multiple testing should be taken into account. Therefore, the BRIEF-P findings should not be interpreted as evidence of broad executive impairment, but rather as subtle variations in everyday functioning that warrant cautious interpretation.

The distinct “trimester-specific” associations observed here likely reflect the dynamic nature of neurodevelopment [30]. The first trimester is a period of intense neurogenesis and neuronal migration [31]. An imbalance of *n*-6/*n*-3 fatty acids during this foundational stage may alter the composition of neuronal membranes, potentially disrupting the formation of fundamental neural circuits that underpin self-regulation [32]. In contrast, the third trimester is dominated by rapid synaptogenesis and myelination [31]. While essential, the developmental processes occurring later in gestation may be more robust to minor fluctuations in fatty acid ratios, or perhaps more reliant on the absolute supply of specific fatty acids rather than their relative balance [33]. Notably, our findings indicate that associations were primarily evident during the first trimester. While prior work highlights the third trimester as a period of rapid DHA accretion supporting structural brain growth, particularly in the frontal lobe and hippocampus, this stage may be more dependent on the absolute supply of specific fatty acids than on their relative balance [34]. In contrast, our results show that associations were mainly observed during early gestation, with implications for executive function and working memory. Consistent with this, we observed a negative association of first-trimester *n*-6/*n*-3 ratios with executive difficulties and working memory index. In the third trimester, however, we observed a positive association with FRI. Notably, the unadjusted positive association observed between third-trimester *n*-6/*n*-3 ratios and fluid reasoning scores (FRI; *p* = 0.026) did not retain statistical significance after correction for multiple testing (FDR-adjusted *p* > 0.05). Given the multiple outcomes and domains evaluated, this isolated observation likely reflects a chance finding rather than a true biological effect, and we have refrained from over-interpreting it. While individual fatty acid ratios (ARA/DHA and ARA/EPA) also demonstrated nominal associations with certain neurodevelopmental domains that were attenuated after FDR adjustment, the global *n*-6/*n*-3 ratio exhibited more persistent associations with executive function subscales after correction. Although this pattern suggests that overall fatty acid balance may be particularly relevant for early executive programming, these exploratory observations should be interpreted with caution and require replication in future well-powered cohorts [12,35]. Our findings suggest that associations with maternal fatty acid balances were predominantly observed during early gestation. However, as fatty acids were quantified at two discrete time points (12 and 36 weeks) rather than across a continuous gestational trajectory, and nominal statistical significance in one trimester does not definitively prove a contrasting effect with another, these timing-specific patterns should be interpreted with caution.

Drawing on the broader experimental and clinical literature, several plausible mechanisms may help contextualize these epidemiological findings, though none were directly measured in this study. Long-chain polyunsaturated fatty acids are essential for neuronal membrane integrity, synaptogenesis, and neurotransmitter function [2,34]. It has been widely hypothesized in prior studies that an *n*-6-skewed maternal profile may shift eicosanoid metabolism toward pro-inflammatory mediators [36], potentially modulating microglial activation and disrupting synaptic pruning, which could theoretically lower executive function development [37]. Furthermore, based on established biochemical principles, enzymatic competition between *n*-6 and *n*-3 may reduce fetal DHA availability, affecting the maturation of the prefrontal cortex—a region critical for inhibition, working memory, and planning [11]. Future mechanistic studies are needed to evaluate whether these pathways underlie the associations observed here.

Our study has several strengths. By measuring maternal fatty acids in both the first and third trimesters, we captured dynamic changes across gestation and identified a critical window of vulnerability in early pregnancy. In addition, we assessed both cognitive abilities and executive functions in preschool-aged children, which are key indicators of early neurodevelopment. These domains are closely linked to later academic performance, behavioral regulation, and overall developmental outcomes, and early identification of deficits provides an opportunity for timely interventions to support optimal child development [38].

Several limitations should be considered when interpreting our findings. First, the study population was drawn from a specific region in Spain, limiting generalizability to other populations with different dietary patterns or genetic backgrounds. Second, executive functions were not comprehensively assessed in the children using dedicated neuropsychological measures. Although cognitive performance was evaluated with the WPPSI, including the WMI as a core component of executive functioning, this construct was otherwise assessed solely through parent-reported measures using the BRIEF-P. Despite their ecological validity, these measures may be subject to informant bias and may not fully reflect performance-based functioning. Third, although we applied the Benjamini–Hochberg FDR correction to account for multiple testing, the large number of exposures and outcomes examined still carries a residual risk of chance findings; nevertheless, the consistency of associations observed across several domains supports the robustness of our primary results. Fourth, exposure was evaluated at two specific gestational time points (12 and 36 weeks). While this design allowed us to explore timing-specific patterns, it does not capture the full gestational trajectory, precluding definitive conclusions regarding a critical developmental window (hence these observations should be interpreted as trimester-specific associations requiring confirmation with formal joint models). Furthermore, although formal interaction tests between maternal fatty-acid ratios and child sex did not reach statistical significance, the possibility of limited statistical power due to our moderate sample size cannot be entirely ruled out when interpreting sex-stratified patterns. Finally, although baseline comparisons showed no significant differences between completers and non-completers, potential selection bias from loss to follow-up over the 4-year period cannot be entirely ruled out, and replication in larger, independent cohorts remains warranted.

## 5. Conclusions

This study found that a higher maternal *n*-6/*n*-3 ratio during the first trimester, but not the third, was associated with poorer executive function in four-year-old children, particularly in working memory, inhibition, and planning. These findings highlight that associations between LCPUFA exposure and child neurodevelopment were primarily observed in the first trimester. However, given the observational nature of this study, these findings do not establish causality or demonstrate that altering maternal dietary fatty-acid intake will directly modify child neurodevelopmental outcomes. Further research, including randomized controlled trials, is needed to confirm these results before translating them into nutritional guidance.

## Figures and Tables

**Figure 1:**
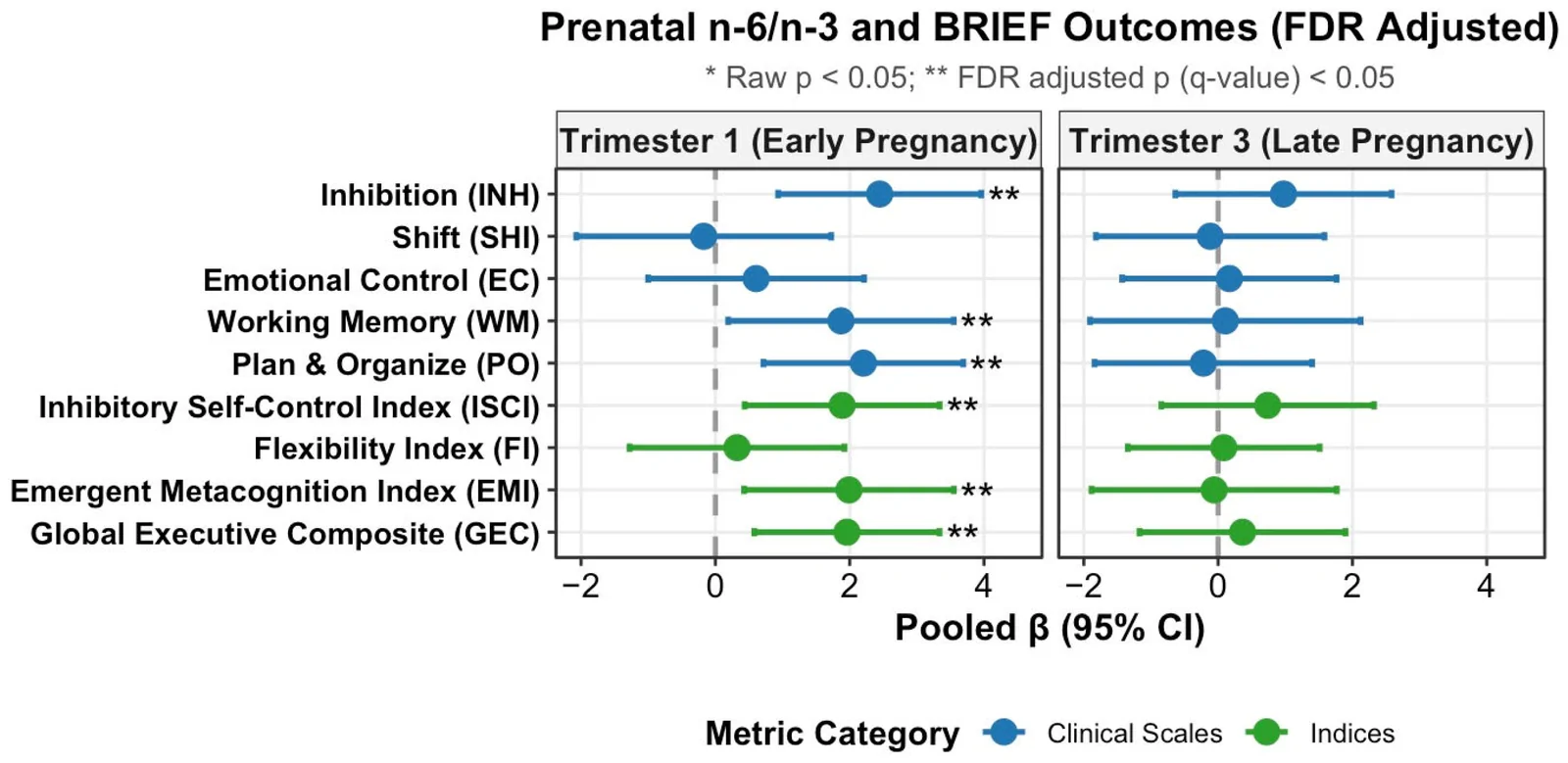
Association between the *n*-6/*n*-3 ratio and Behavior Rating Inventory of Executive Function—Preschool Version scores. Values are regression coefficients and 95% CIs representing the change in BRIEF scores per 1-SD increase in the natural log-transformed maternal *n*-6/*n*-3 ratio, adjusted for maternal age, 1st- and 3rd-trimester BMI, social class, smoking status, Mediterranean diet score, gestational age, and child sex.

**Figure 2:**
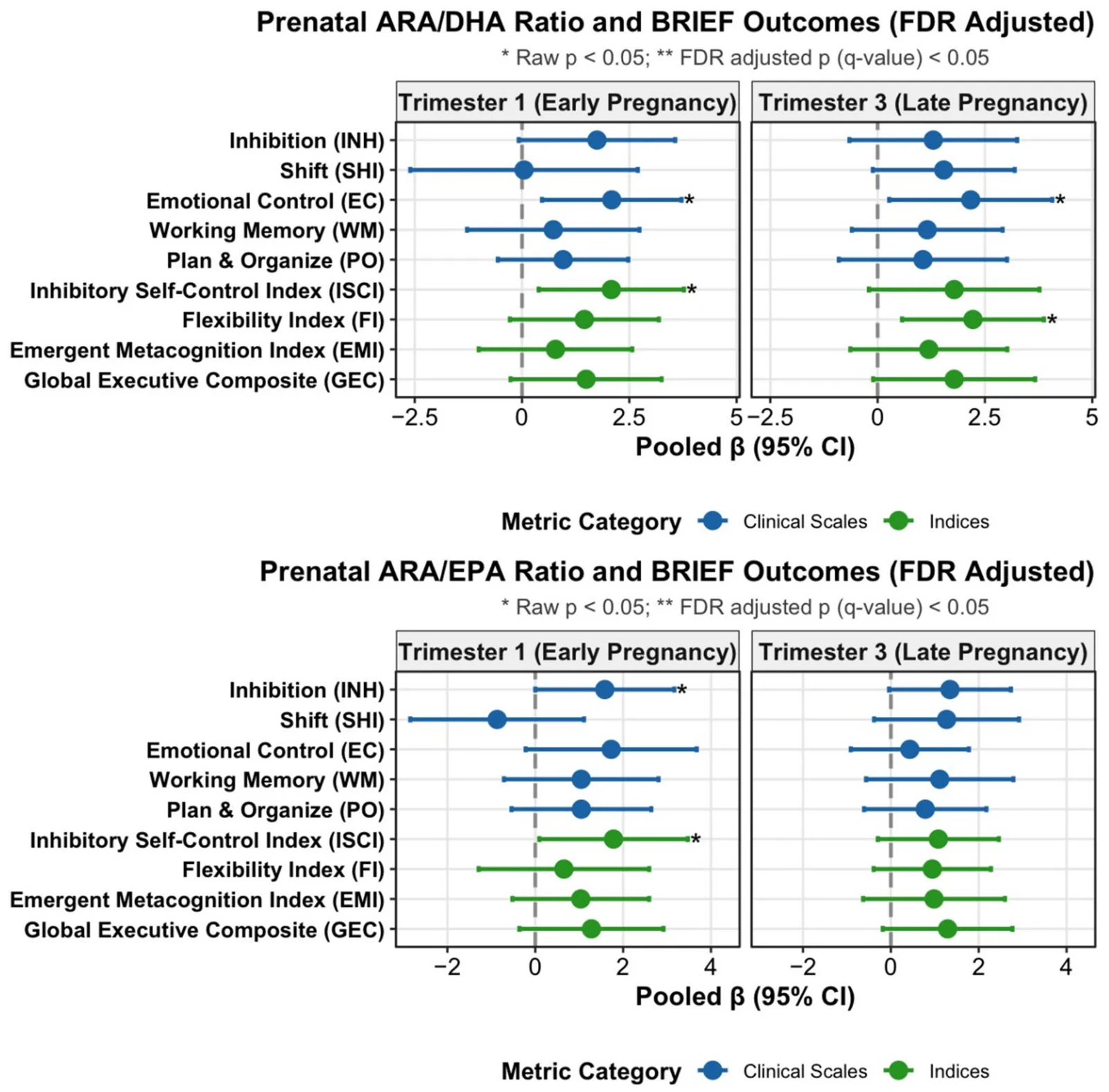
Associations of ARA/DHA and ARA/EPA ratios with Behavior Rating Inventory of Executive Function–Preschool Version scores. Values are regression coefficients and 95% CIs representing the change in BRIEF scores per 1-SD increase in the natural log-transformed maternal ARA/DHA and ARA/EPA ratio, adjusted for maternal age, 1st- and 3rd-trimester BMI, social class, smoking status, Mediterranean diet score, gestational age, and child sex.

**Figure 3:**
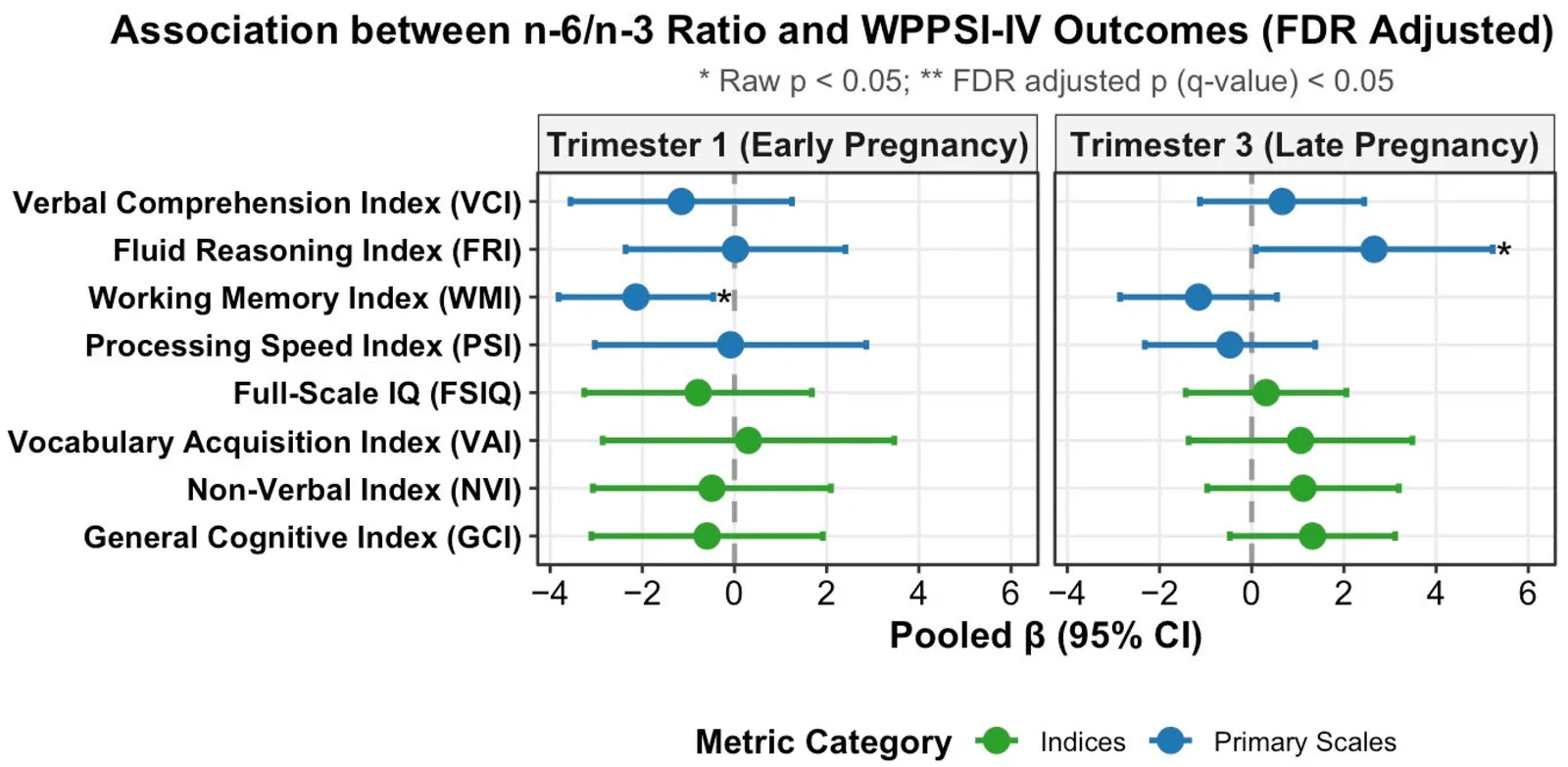
Association between the *n*-6/*n*-3 ratio with Wechsler Preschool and Primary Scale of Intelligence scores. Values are regression coefficients and 95% CIs representing the change in WPPSI-IV scores per 1-SD increase in the natural log-transformed maternal *n*-6/*n*-3 ratio, adjusted for maternal age, 1st- and 3rd-trimester BMI, social class, smoking status, Mediterranean diet score, gestational age, child sex, and parental IQ.

**Figure 4:**
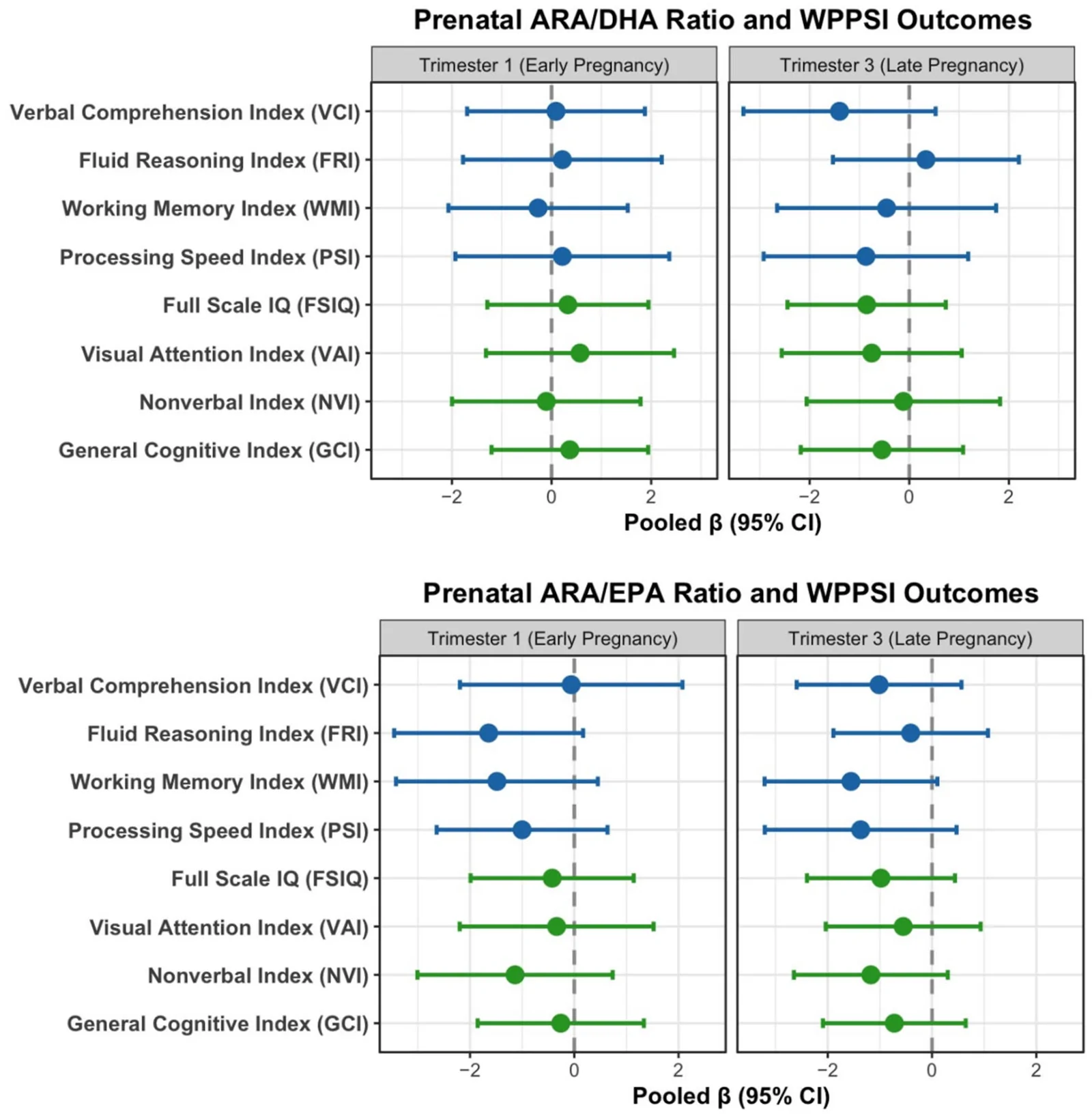
Associations of ARA/DHA and ARA/EPA ratios with Wechsler Preschool and Primary Scale of Intelligence scores. Values are regression coefficients and 95% CIs representing the change in WPPSI-IV scores per 1-SD increase in the natural log-transformed maternal ARA/DHA and ARA/EPA ratio, adjusted for maternal age, 1st- and 3rd-trimester BMI, social class, smoking status, Mediterranean diet score, gestational age, child sex, and parental IQ.

**Table 1:** General characteristics of mother and offspring (*n* = 223).

Mother Characteristics	1st Trimester	3rd Trimester	*P*	Missing *n* (%)
ARA/DHA, median (IQR)	3.46 (0.88)	3.13 (0.87)	<0.001	0 (0.0%)
ARA/EPA, median (IQR)	27.38 (17.21)	38.08 (21.55)	<0.001	0 (0.0%)
*n*-6/*n*-3, median (IQR)	15.44 (4.69)	29.33 (11.12)	<0.001	0 (0.0%)
Age (mean (SD))	40.14 (4.29)			0 (0.0%)
BMI (mean (SD)) *	25.13 (4.64)	29.20 (4.38)		0 (0.0%)
Social class (%)				6 (2.7%)
Low	53 (23.8)			
Middle	86 (38.6)			
High	84 (37.7)			
Smoke status (%)				
Smoker	31 (13.9)			
No-smoker	192 (86.1)			
MedDiet score (mean (SD))	9.59 (2.57)	9.94 (2.52)	0.054	6 (2.7%)
Children and follow-up characteristics				
Gestation age, weeks (mean (SD))	39.94 (1.14)			0 (0.0%)
Child sex (%)				0 (0.0%)
male	118 (52.9)			
female	105 (47.1)			
Type of feeding (%)				0 (0.0%)
Mixed feeding/infant formula	46 (20.6)			
Breastfeeding	177 (79.4)			
SDQI score at age four (mean (SD))	61.78 (10.17)			5 (2.2%)
Parental IQ (mean (SD))	9.09 (3.61)			9 (4.0%)

Abbreviation: *n*-6/*n*-3, Omega-6 to Omega-3 fatty acid ratio; BMI, Body Mass Index; MedDiet, Mediterranean Diet; IQ, Intelligence Quotient; SDQI, Spanish Diet Quality Index; SD, Standard Deviation; IQR, Interquartile Range. *, Maternal BMI in the 1st and 3rd trimesters are presented as descriptive characteristics at each respective time point; statistical comparisons between trimesters were not performed given normal gestational weight progression. Data are presented as mean (SD) for normally distributed variables, median (IQR) for non-normally distributed variables (including fatty-acid ratios), and *n* (%) for categorical variables.

**Table 2:** Neurodevelopmental scales in children: mean BRIEF-P T-scores and WPPSI-IV composite scores.

	Overall	Male	Female	
BRIEF-P scales	mean (SD)	mean (SD)	mean (SD)	*p*
Inhibition	50.18 (10.07)	52.71 (11.02)	46.95 (7.32)	<0.001
Shift	50.84 (10.08)	50.27 (9.65)	50.87 (9.60)	0.704
Emotional Control	49.63 (10.34)	51.30 (12.58)	48.17 (7.66)	0.067
Working Memory	50.74 (10.03)	52.17 (9.42)	47.28 (8.69)	0.001
Planning/Organization	50.30 (9.65)	51.81 (9.41)	47.16 (7.97)	0.001
Inhibitory Self-Control Index	49.94 (10.04)	52.29 (11.31)	47.16 (7.17)	0.001
Flexibility Index	50.26 (9.90)	51.04 (10.83)	49.33 (8.50)	0.283
Emergent Metacognition Index	50.50 (9.91)	52.16 (9.38)	46.97 (8.41)	<0.001
Global Executive Composite	50.40 (9.89)	52.27 (10.41)	47.24 (7.26)	0.001
WPPSI scales				
Verbal Comprehension Index	104.93 (13.35)	103.36 (15.03)	108.64 (11.68)	0.008
Fluid Reasoning Index	102.69 (13.13)	101.69 (13.09)	104.59 (12.86)	0.121
Working Memory Index	98.01 (12.11)	96.98 (12.14)	99.74 (11.34)	0.104
Processing Speed Index	95.71 (12.63)	92.05 (12.06)	100.11 (11.51)	<0.001
Full-Scale Intelligence Quotient	102.25 (11.68)	100.34 (12.18)	105.55 (10.42)	0.002
Vocabulary Acquisition Index	97.49 (14.00)	94.36 (14.88)	101.45 (12.80)	0.001
Nonverbal Index	101.16 (12.24)	99.43 (12.37)	103.41 (10.92)	0.019
General Cognitive Index	105.93 (12.11)	104.87 (13.46)	108.27 (10.59)	0.054

Abbreviation: BRIEF-P, Behavior Rating Inventory of Executive Function—Preschool Version; WPPSI, Wechsler Preschool and Primary Scale of Intelligence.

## Data Availability

The data presented in this study are available on request from the corresponding author. The data are not publicly available due to privacy and ethical restrictions.

## Supplementary Materials

The following supporting information can be downloaded at: https://www.mdpi.com/article/10.3390/nu18213514/s1, Figure S1. Study flow; Figure S2. Association between the *n*-6/*n*-3 ratio and Behavior Rating Inventory of Executive Function—Preschool Version scores. Values are regression coefficients and 95% CIs representing the change in BRIEF scores per 1-SD increase in the natural log-transformed maternal *n*-6/*n*-3 ratio, adjusted for maternal age, 1st- and 3rd-trimester BMI, social class, smoking status, Mediterranean diet score, gestational age, child sex, type of feeding, and SDQI score at age four; Figure S3. Associations of ARA/DHA and ARA/EPA ratios with Behavior Rating Inventory of Executive Function–Preschool Version scores. Values are regression coefficients and 95% CIs representing the change in BRIEF scores per 1-SD increase in the natural log-transformed maternal ARA/DHA and ARA/EPA ratio, adjusted for maternal age, 1st- and 3rd-trimester BMI, social class, smoking status, Mediterranean diet score, gestational age, child sex, type of feeding, and SDQI score at age four; Figure S4. Association between the *n*-6/*n*-3 ratio and Wechsler Preschool and Primary Scale of Intelligence scores. Values are regression coefficients and 95% CIs representing the change in WPPSI-IV scores per 1-SD increase in the natural log-transformed maternal *n*-6/*n*-3 ratio, adjusted for maternal age, 1st- and 3rd-trimester BMI, social class, smoking status, Mediterranean diet score, gestational age, child sex, type of feeding, parental IQ, and SDQI score at age four; Figure S5. Associations of ARA/DHA and ARA/EPA ratios with Wechsler Preschool and Primary Scale of Intelligence scores. Values are regression coefficients and 95% CIs representing the change in WPPSI-IV scores per 1-SD increase in the natural log-transformed maternal ARA/DHA and ARA/EPA ratio, adjusted for maternal age, 1st- and 3rd-trimester BMI, social class, smoking status, Mediterranean diet score, gestational age, child sex, type of feeding, parental IQ, and SDQI score at age four; Table S1. Comparison of characteristics between included and excluded participants; Table S2. Interaction analyses between maternal *n*-6/*n*-3 ratios and child sex on WPPSI-IV and BRIEF-P outcomes; Table S3. Association between prenatal *n*-6/*n*-3 ratio and BRIEF scales; Table S4. Association between prenatal ARA/DHA ratio and BRIEF scales; Table S5. Association between prenatal ARA/EPA ratio and BRIEF scales; Table S6. Association between prenatal *n*-6/*n*-3 ratio and WPPSI scales; Table S7. Association between prenatal ARA/DHA ratio and WPPSI scales; Table S8. Association between prenatal ARA/EPA ratio and WPPSI scales.
