# Supplementary material for: Trimester-Specific Associations Between Maternal Fatty Acid Ratios and Cognitive and Executive Functioning in 4-Year-Old Children
Source: Nutrients. 2026 Oct 26;18(21):3514. doi: 10.3390/nu18213514 (PMC13764854; doi:10.3390/nu18213514)
Supplement: Supplementary file 1 [file nutrients-18-03514-s001.zip › nutrients-4575637-supplementary.pdf]

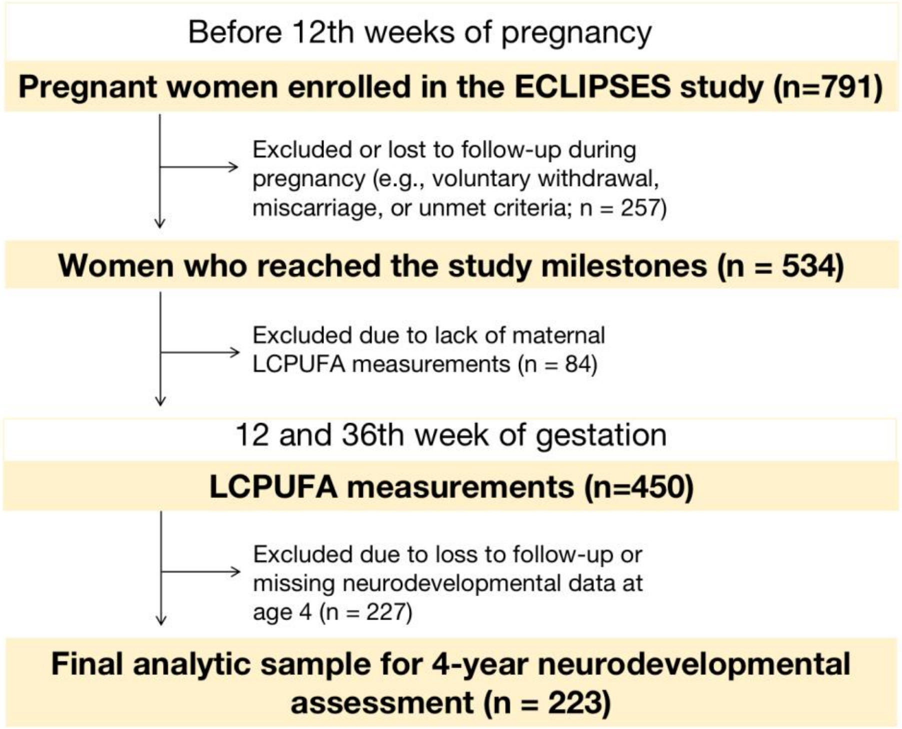

Figure S1. Study flow

Supplementary Methods: Detailed Protocol for Long-Chain Fatty Acid Analysis

Blood samples were collected at the first and third trimesters of pregnancy. They were collected in one 7.5 mL tube without anticoagulant and were left unmixed for 30 min at room temperature to allow coagulation. The serum was centrifuged and distributed in aliquots of 500 µL and stored at −80 °C in the BioBank. At the end of the clinical study, samples were thawed and processed simultaneously in order to minimize inter-batch variation.Fatty acids (FAs) such as saturated (SFAs), monounsaturated (MUFAs), and polyunsaturated fatty acids (PUFAs) were analyzed. A gas chromatography–mass spectrometry (GC-MS) system using a 7890A GC equipment coupled to a QqQ 7000 Series® (Agilent Technologies Inc., Santa Clara, USA) was used after their derivatization to methyl esters (FAMEs) due to their higher volatility. A volume of 50 µL of serum samples was blended with an internal standard (IS) solution (Myristic d-27 acid, Merck KGaA, Darmstadt, Germany), chloroform, and methanolic hydrochloric acid, and incubated at 80 °C for 2 hours. Then, FAMEs were extracted by liquid-liquid extraction using hexane and injected into the GC-MS system.Chromatographic analysis was carried out to determine the FAMEs included in the Food Industry FAME Mix (Restek Corporation, Pennsylvania, USA). FAMEs were separated on a high-polarity column (100 m × 250 µm × 0.25 µm; HP-88 column, Agilent Technologies Inc., USA) with a temperature program between 140 and 240 °C at 1 mL/min using helium as the carrier gas. Ionization was performed by electron impact (70 eV), and the mass analyzer

operated in Selected Ion Monitoring (SIM) mode.The quantified FAs included:SFAs: lauric acid, myristic acid, palmitic acid, and stearic acid (summed to obtain total SFA).MUFAs: palmitoleic acid and oleic acid (summed to obtain total MUFA).n-6 PUFAs: linoleic acid (LA), dihomo-gamma-linolenic acid, and arachidonic acid (AA) (summed to obtain total n-6 PUFA).n-3 PUFAs: eicosapentaenoic acid (EPA) and docosahexaenoic acid (DHA) (summed to obtain total n-3 PUFA).Total long-chain fatty acids were calculated as the sum of total SFA + total MUFA + total n-6 PUFA + total n-3 PUFA.

Supplementary Table S1. Comparison of characteristics between included and excluded participants

Mother characteristics	Included	Excluded	p
Age (mean (SD))	40.14 (4.29)	40.25 (4.35)	0.482
BMI 1st trimester (mean (SD)	25.13 (4.64)	24.85 (4.50)	0.512
BMI 3rd trimester (mean (SD)	29.20 (4.38)	28.95 (4.20)	0.543
Social class (%)			0.715
Low	53 (23.8)	60 (26.4)	
Middle	86 (38.6)	87 (38.3)	
High	84 (37.7)	80 (35.2)	
Smoke status (%)			0.652
Smoker	31 (13.9)	35 (15.4)	
No-smoker	192 (86.1)	192 (84.6)	
MedDiet score (mean (SD))	9.59 (2.57)	9.45 (2.50)	0.054
Children and follow-up characteristics			
Gestation age, weeks (mean (SD))	39.94 (1.14)	39.88 (1.10)	0.581
Child sex (%)			0.674
male	118 (52.9)	115 (50.7)	
female	105 (47.1)	112 (49.3)	
Type of feeding (%)			0.871
Mixed feeding/infant formula	46 (20.6)	48 (21.1)	
Breastfeeding	177 (79.4)	179 (78.9)	
SDQI score at age four (mean (SD))	61.78 (10.17)	-	-
Parental IQ (mean (SD))	9.09 (3.61)	8.95 (3.70)	0.684

Abbreviation: BMI, Body Mass Index; MedDiet, Mediterranean Diet; IQ, Intelligence Quotient; SDQI, Spanish Diet Quality Index; SD, Standard Deviation

Supplementary table S2. Interaction analyses between maternal n-6/n-3 ratios and child sex on WPPSI-IV and BRIEF-P outcomes

Outcome	Trimester	Estimate (SE)	P-interaction
VCI	T1	-1.25 (1.68)	0.459
	T3	0.01 (1.79)	0.997
FRI	T1	0.79 (2.07)	0.707
	T3	1.72 (1.73)	0.32
WMI	T1	-1.37 (1.74)	0.432
	T3	0.05 (1.75)	0.976

PSI	T1	-2.10 (1.64)	0.202
	T3	-0.71 (1.72)	0.682
FSIQ	T1	-1.03 (1.64)	0.535
	T3	-0.05 (1.45)	0.972
VAI	T1	-1.55 (1.87)	0.41
	T3	-0.89 (1.80)	0.623
NVI	T1	-0.56 (1.80)	0.755
	T3	0.20 (1.72)	0.906
GCI	T1	-0.43 (1.58)	0.787
	T3	-0.07 (1.48)	0.964
INH	T1	1.06 (1.50)	0.485
	T3	1.33 (1.65)	0.429
SHI	T1	2.79 (1.93)	0.169
	T3	0.97 (1.80)	0.598
EC	T1	1.45 (1.68)	0.395
	T3	1.04 (1.55)	0.502
WM	T1	0.52 (1.47)	0.727
	T3	-0.24 (1.50)	0.875
PO	T1	1.86 (1.21)	0.126
	T3	0.96 (1.60)	0.553
ISCI	T1	1.31 (1.53)	0.397
	T3	1.31 (1.53)	0.397
FI	T1	2.45 (1.71)	0.169
	T3	1.17 (1.54)	0.449
EMI	T1	1.09 (1.32)	0.412
	T3	0.20 (1.57)	0.901
GEC	T1	1.71 (1.41)	0.233
	T3	0.97 (1.53)	0.531

WPPSI, Wechsler Preschool and Primary Scale of Intelligence; VCI, Verbal Comprehension Index; FRI, Fluid Reasoning Index; WMI, Working Memory Index; PSI, Processing Speed Index; FSIQ, Full Scale Intelligence Quotient; VAI, Vocabulary Acquisition Index; NVI, Nonverbal Index; GCI, General Cognitive Index. BRIEF, Behavior Rating Inventory of Executive Function; INH, Inhibit; SHI, Shift; EC, Emotional Control; WM, Working Memory; PO, Plan/Organize; ISCI, Inhibitory Self-Control Index; FI, Flexibility Index; EMI, Emergent Metacognition Index; GEC, Global Executive Composite.

Supplementary Table S3. Association between prenatal n-6/n-3 ratio and BRIEF scales

BRIEF scales	1st trimester			3rd trimester	
	b (95%CI)	p	p_fdr	b (95%CI)	p
INH	2.45 (0.93,	0.002	0.017	0.97 (-0.64,	0.228

	3.96)			2.58)	
SHI	-0.17 (-2.07, 1.72)	0.853	0.853	-0.12 (-1.82, 1.58)	0.89
EC	0.61 (-1.00, 2.21)	0.456	0.586	0.17 (-1.43, 1.76)	0.835
WM	1.87 (0.19, 3.55)	0.03	0.045	0.11 (-1.91, 2.12)	0.911
PO	2.20 (0.72, 3.69)	0.004	0.017	-0.22 (-1.84, 1.40)	0.782
ISCI	1.89 (0.44, 3.34)	0.011	0.025	0.74 (-0.84, 2.32)	0.352
FI	0.32 (-1.28, 1.92)	0.689	0.775	0.08 (-1.34, 1.51)	0.907
EMI	1.99 (0.43, 3.55)	0.014	0.025	-0.06 (-1.89, 1.77)	0.946
GEC	1.96 (0.58, 3.34)	0.006	0.017	0.36 (-1.17, 1.90)	0.634

Abbreviation: n-6, omega-6; n-3, omega-3; BRIEF, Behavior Rating Inventory of Executive Function; INH, Inhibit; SHI, Shift; EC, Emotional Control; WM, Working Memory; PO, Plan/Organize; ISCI, Inhibitory Self-Control Index; FI, Flexibility Index; EMI, Emergent Metacognition Index; GEC, Global Executive Composite.

Supplementary Table S4. Association between prenatal ARA/DHA ratio and BRIEF scales

BRIEF scales	1st trimester		3rd trimester	
	b (95%CI)	p	b (95%CI)	p
INH	1.75 (-0.08, 3.57)	0.06	1.30 (-0.66, 3.25)	0.179
SHI	0.05 (-2.60, 2.70)	0.969	1.54 (-0.11, 3.19)	0.067
EC	2.09 (0.47, 3.72)	0.012	2.17 (0.27, 4.07)	0.027
WM	0.73 (-1.28, 2.74)	0.454	1.16 (-0.60, 2.91)	0.189
PO	0.96 (-0.56, 2.48)	0.212	1.06 (-0.90, 3.02)	0.269
ISCI	2.08 (0.39, 3.77)	0.017	1.78 (-0.20, 3.77)	0.075
FI	1.45 (-0.28, 3.19)	0.099	2.22 (0.57, 3.87)	0.009
EMI	0.78 (-1.01, 2.57)	0.376	1.19 (-0.64, 3.02)	0.189
GEC	1.49 (-0.27, 3.25)	0.093	1.78 (-0.10, 3.67)	0.062

Abbreviation: DHA, docosahexaenoic acid; ARA, arachidonic acid; BRIEF, Behavior Rating Inventory of Executive Function; INH, Inhibit; SHI, Shift; EC, Emotional Control; WM, Working Memory; PO, Plan/Organize; ISCI, Inhibitory Self-Control Index; FI, Flexibility Index; EMI, Emergent Metacognition Index; GEC, Global Executive Composite.

Supplementary Table S5. Association between prenatal ARA/EPA ratio and BRIEF scales

BRIEF scales	1st trimester		3rd trimester	
	b (95%CI)	p	b (95%CI)	p
INH	1.58 (0.00, 3.17)	0.05	1.35 (-0.04, 2.73)	0.057
SHI	-0.87 (-2.85, 1.11)	0.378	1.27 (-0.38, 2.92)	0.124
EC	1.73 (-0.22, 3.67)	0.08	0.43 (-0.91, 1.78)	0.524
WM	1.05 (-0.71, 2.81)	0.237	1.11 (-0.56, 2.79)	0.177
PO	1.05 (-0.54, 2.64)	0.192	0.78 (-0.61, 2.18)	0.258
ISCI	1.78 (0.09, 3.47)	0.039	1.08 (-0.30, 2.46)	0.12
FI	0.65 (-1.29, 2.59)	0.498	0.94 (-0.39, 2.28)	0.162
EMI	1.04 (-0.52, 2.59)	0.189	0.98 (-0.63, 2.60)	0.215
GEC	1.28 (-0.36, 2.92)	0.124	1.29 (-0.18, 2.77)	0.083

Abbreviation: ARA, arachidonic acid; EPA, eicosapentaenoic acid; BRIEF, Behavior Rating Inventory of Executive Function; INH, Inhibit; SHI, Shift; EC, Emotional Control; WM, Working Memory; PO, Plan/Organize; ISCI, Inhibitory Self-Control Index; FI, Flexibility Index; EMI, Emergent Metacognition Index; GEC, Global Executive Composite.

Supplementary Table S6. Association between prenatal n-6/n-3 ratio and WPPSI scales

WPPSI scales	1st trimester		3rd trimester	
	b (95%CI)	p	b (95%CI)	p
VCI	-1.16 (-3.56, 1.25)	0.323	0.66 (-1.12, 2.44)	0.465
FRI	0.02 (-2.36, 2.40)	0.986	2.66 (0.08, 5.23)	0.044
WMI	-2.14 (-3.82, -0.46)	0.013	-1.15 (-2.86, 0.55)	0.181
PSI	-0.09 (-3.03, 2.86)	0.949	-0.47 (-2.32, 1.38)	0.611
FSIQ	-0.79 (-3.26, 1.68)	0.496	0.31 (-1.43, 2.05)	0.72
VAI	0.30 (-2.86, 3.46)	0.834	1.06 (-1.37, 3.48)	0.369
NVI	-0.49 (-3.07, 2.09)	0.686	1.11 (-0.97, 3.19)	0.279
GCI	-0.59 (-3.11, 1.92)	0.614	1.32 (-0.47, 3.11)	0.145

Abbreviation: n-6, omega-6; n-3, omega-3; WPPSI, Wechsler Preschool and Primary Scale of Intelligence; VCI, Verbal Comprehension Index; FRI, Fluid Reasoning Index; WMI, Working Memory Index; PSI, Processing Speed Index; FSIQ, Full Scale Intelligence Quotient; VAI, Vocabulary Acquisition Index; NVI, Nonverbal Index; GCI, General Cognitive Index.

Supplementary Table S7. Association between prenatal ARA/DHA ratio and WPPSI scales

WPPSI scales	1st trimester		3rd trimester	
	b (95%CI)	p	b (95%CI)	p
VCI	0.09 (-1.70, 1.87)	0.922	-1.40 (-3.33, 0.53)	0.161
FRI	0.22 (-1.78, 2.21)	0.832	0.33 (-1.53, 2.20)	0.727
WMI	-0.27 (-2.07, 1.53)	0.77	-0.46 (-2.65, 1.74)	0.69
PSI	0.22 (-1.93, 2.36)	0.846	-0.87 (-2.92, 1.18)	0.413
FSIQ	0.33 (-1.29, 1.95)	0.693	-0.86 (-2.44, 0.73)	0.293
VAI	0.57 (-1.32, 2.46)	0.556	-0.75 (-2.56, 1.05)	0.415
NVI	-0.11 (-2.00, 1.79)	0.913	-0.12 (-2.06, 1.82)	0.905
GCI	0.37 (-1.21, 1.94)	0.648	-0.55 (-2.18, 1.08)	0.509

Abbreviation: DHA, docosahexaenoic acid; ARA, arachidonic acid; WPPSI, Wechsler Preschool and Primary Scale of Intelligence; VCI, Verbal Comprehension Index; FRI, Fluid Reasoning Index; WMI, Working Memory Index; PSI, Processing Speed Index; FSIQ, Full Scale Intelligence Quotient; VAI, Vocabulary Acquisition Index; NVI, Nonverbal Index; GCI, General Cognitive Index.

Supplementary Table S8. Association between prenatal ARA/EPA ratio and WPPSI scales

WPPSI scales	1st trimester		3rd trimester	
	b (95%CI)	p	b (95%CI)	p
VCI	-0.06 (-2.19, 2.08)	0.957	-1.01 (-2.60, 0.57)	0.214
FRI	-1.64 (-3.46, 0.17)	0.078	-0.41 (-1.89, 1.07)	0.589
WMI	-1.48 (-3.42, 0.45)	0.143	-1.55 (-3.21, 0.10)	0.077
PSI	-1.00 (-2.64, 0.64)	0.232	-1.37 (-3.21, 0.47)	0.163
FSIQ	-0.42 (-1.99, 1.14)	0.596	-0.98 (-2.40, 0.44)	0.183
VAI	-0.34 (-2.20, 1.52)	0.723	-0.55 (-2.04, 0.93)	0.467
NVI	-1.14 (-3.01, 0.74)	0.241	-1.17 (-2.65, 0.30)	0.123
GCI	-0.26 (-1.85, 1.34)	0.751	-0.72 (-2.09, 0.65)	0.303

Abbreviation: EPA, eicosapentaenoic acid; ARA, arachidonic acid; WPPSI, Wechsler Preschool and Primary Scale of Intelligence; VCI, Verbal Comprehension Index; FRI, Fluid Reasoning Index; WMI, Working Memory Index; PSI, Processing Speed Index; FSIQ, Full Scale Intelligence Quotient; VAI, Vocabulary Acquisition Index; NVI, Nonverbal Index; GCI, General Cognitive Index.

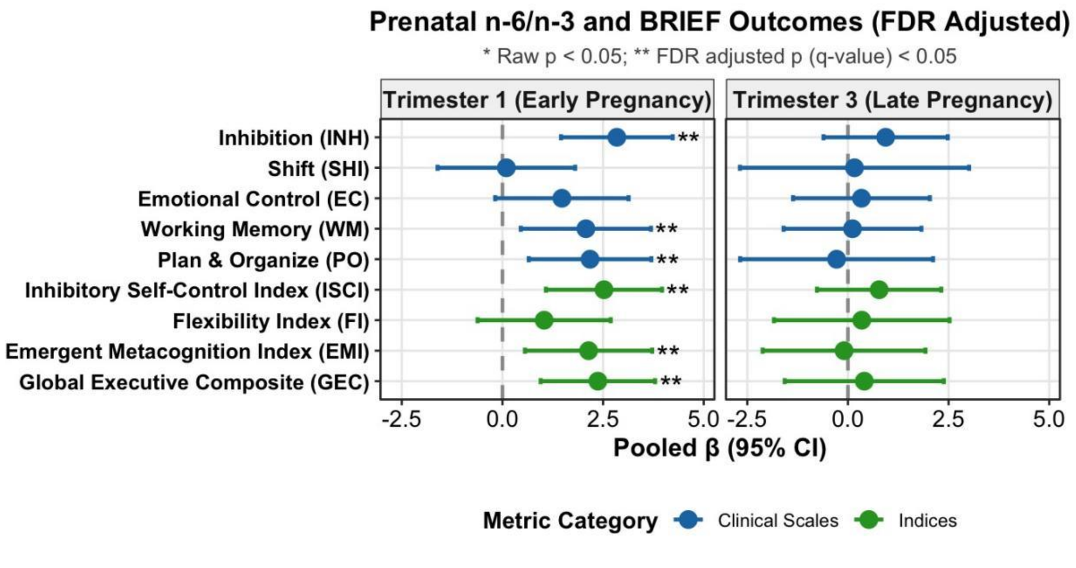

Figure S2 Association between the n-6/n-3 ratio and Behavior Rating Inventory of Executive Function – Preschool Version scores. Values are regression coefficients and 95% CIs representing the change in BRIEF scores per 1-SD increase in the natural log-transformed maternal n-6/n-3 ratio, adjusted for maternal age, 1st and 3rd trimester BMI, social class, smoking status, Mediterranean diet score, gestational age, child sex, type of feeding, and SDQI score at age four.

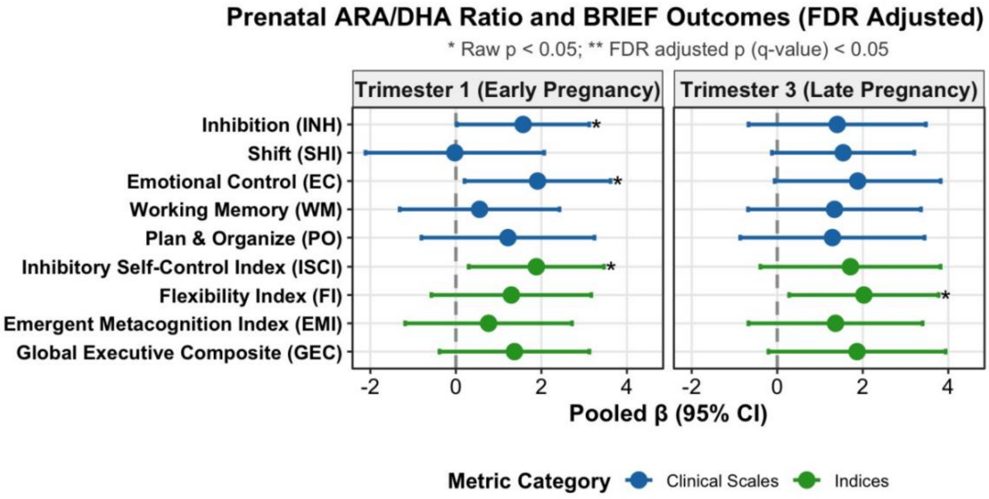

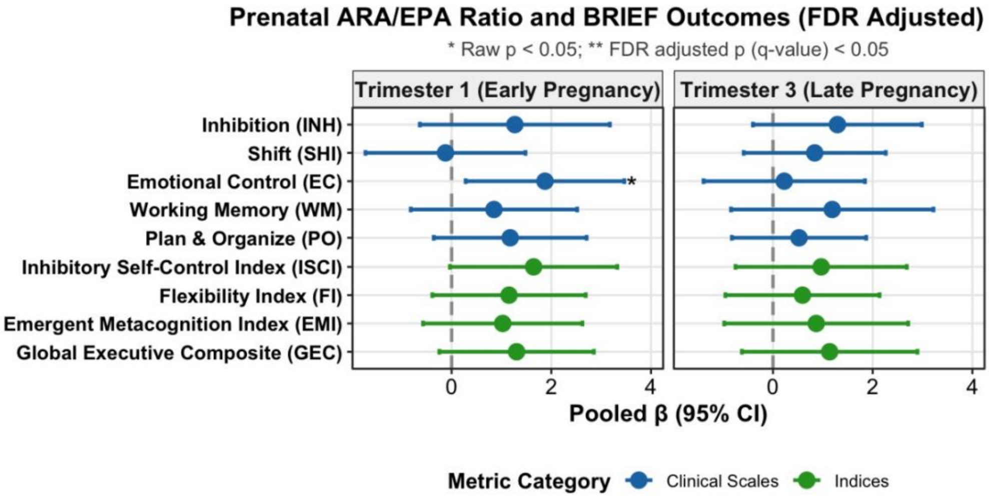

Figure S3 Associations of ARA/DHA and ARA/EPA ratios with Behavior Rating Inventory of Executive Function –Preschool Version scores. Values are regression coefficients and 95% CIs representing the change in BRIEF scores per 1-SD increase in the natural log-transformed maternal ARA/DHA and ARA/EPA ratio, adjusted for maternal age, 1st and 3rd trimester BMI, social class, smoking status, Mediterranean diet score, gestational age, child sex, type of feeding, and SDQI score at age four.

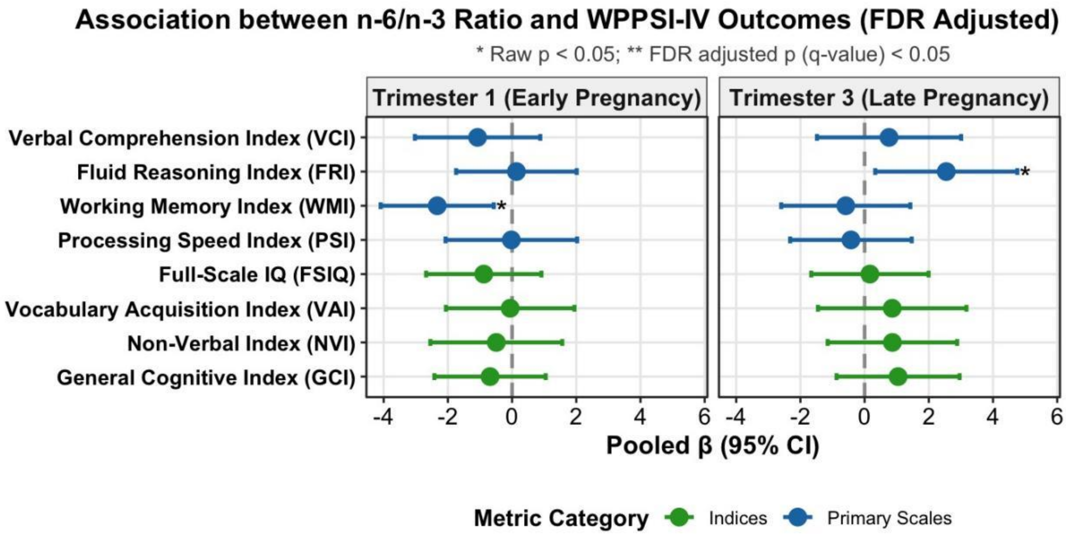

Figure S4 Association between the n-6/n-3 ratio and Wechsler Preschool and Primary Scale of Intelligence scores. Values are regression coefficients and 95% CIs representing the change in WPPSI-IV scores per 1-SD increase in the natural log-transformed maternal n-6/n-3 ratio, adjusted for maternal age, 1st and 3rd trimester BMI, social class, smoking status, Mediterranean diet score, gestational age, child sex, type of feeding, parental IQ, and SDQI score at age four.

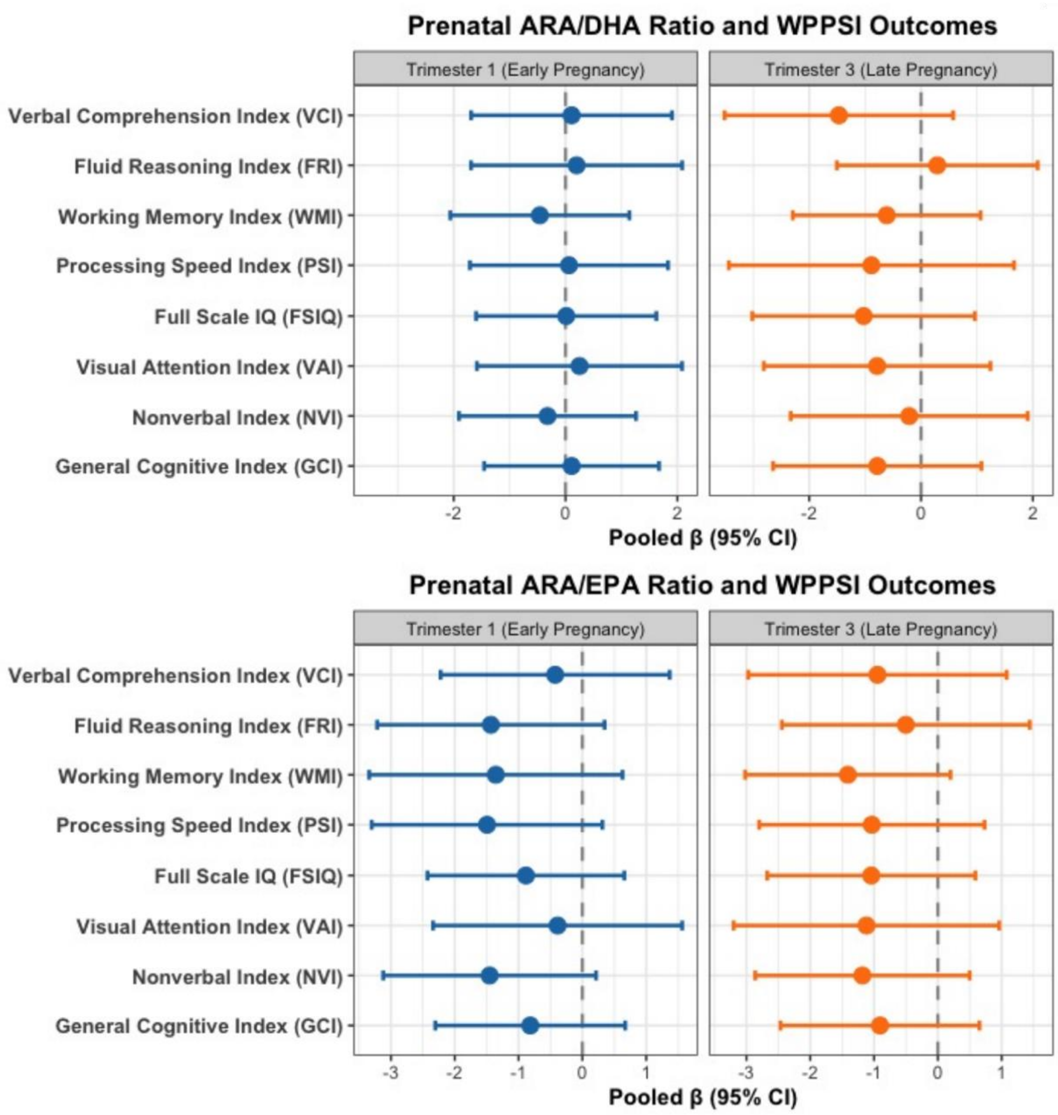

Figure S5 Associations of ARA/DHA and ARA/EPA ratios with Wechsler Preschool and Primary Scale of Intelligence scores. Values are regression coefficients and 95% CIs representing the change in WPPSI-IV scores per 1-SD increase in the natural log-transformed maternal ARA/DHA and ARA/EPA ratio, adjusted for maternal age, 1st and 3rd trimester BMI, social class, smoking status, Mediterranean diet score, gestational age, child sex, type of feeding, parental IQ, and SDQI score at age four.
